# An Analysis of Complaints in Two Large Tertiary University Teaching Hospital ENT Departments: A Two-Year Retrospective Review

**DOI:** 10.1155/2020/1484687

**Published:** 2020-03-18

**Authors:** Iulia Bujoreanu, Ahmad Hariri, Vikas Acharya, Ali Taghi

**Affiliations:** ^1^Imperial College London, London, UK; ^2^St. Mary's Hospital, London, UK

## Abstract

*Introduction*. Complaints relating to patient care are known to correlate with surgical complication rates and malpractice lawsuits. In a continually evolving health service and on-going financial pressures, identifying current complaint themes could drive future improvements in healthcare delivery.

**Objective:**

The aim of this paper is to review and analyse complaints received by the ENT department of two large teaching hospitals in London in order to determine current trends and mitigate future challenges.

**Method:**

All complaints registered with the Patient Advice and Liaison Service (PALS) from the ENT Department at our institution were collected between June 2016 and August 2018. Demographic information was collated and complaints were analysed and interpreted as per a standardised coding taxonomy.

**Results:**

A total of 242 complaints were collected. Most (91.7%) were logged by patients themselves with a mean age of 48.3 (range 3–98 years). The majority were directed at the administrative team (52%) followed by management (23.5%) and then clinicians (16.9%). Administrative issues were the most common (50.1%) followed by clinical (25.1%) and relationship/communication (24.7%). The bulk of complaints focused on delays in access to services and treatment in the form of cancellations and long appointment waiting times (37%).

**Conclusion:**

There has been a significant shift in complaints themes from clinical issues to administrative issues. This may reflect increasing financial and staffing pressures in the NHS. Complaints analysis is key in quality improvement and a cross-specialty integrated filing system in concordance with the recently proposed taxonomy would ease future collection and analysis of data.

## 1. Introduction

There has recently been a cultural shift in managing healthcare complaints, with an increased focus on patient experience as an identifier of quality in delivery of care. This comes as no surprise, as complaints about the standard of patient care have been shown to correlate with the rate of surgical complications and are directly linked to malpractice lawsuits and compensation claims [1, 2]. Good healthcare encounters for patients require good communication, adequate information, and having the opportunity to be involved in the decision-making process. This strengthens the patient-doctor relationship and can increase patient adherence to medical treatment [3]. Therefore, analysing patient complaints and identifying recurring themes is a key step in identifying areas of improvement and enhancing quality of care.

Whilst previous studies have focused on the doctor-patient relationship with poor communication being a key contributor to complaints, it remains unclear as to what role the healthcare provider as a whole has to patient satisfaction. Patients see a good healthcare encounter as one that focuses on their needs, with good communication and having input into the decision-making process, which evidentiates a shift from the traditional paternalistic pattern of care [4].

Additionally, with a continually evolving national health service and on-going financial pressures, identifying current determinants for patient complains is needed to drive further improvement and to recognise and address other contributory factors [5].

Lack of standardised taxonomy and collection of complaints has made extrapolating data difficult, with questionable applicability. However, a recent taxonomy published in the BMJ by Reader et al. offers a comprehensive overview of complaint categories and paves the way for future systematic reviews [5]. Furthermore, there has been a recent shift in thinking, and patient experience is gaining more weight as a marker of quality of care. As hospital care has become increasingly subspecialised and fragmented, patients' views can offer an overall assessment of system efficiency [6]. Subjective patient perception of care correlates to the objective measurements of safety and quality of care, both in the medical and surgical settings. Hospitals which patients scored higher, or which they were more likely to recommend, were shown to have lower rates of complications, medication errors, or discharge complications [7].

We aim to review and analyse complaints received in the Ear, Nose, and Throat Department across two sites of a large university teaching hospital in London over a 2-year period in order to determine current issues and describe how trends in patient complaints have changed. This will provide insight into what steps could be taken to address these and therefore further improve quality of care.

## 2. Methods

All formal and informal complaints registered with the Patient Advice and Liaison Service (PALS) from the ENT Department at our institution were collected between June 2016 and August 2018. Demographic information was collated and complaints were analysed and interpreted as per the coding taxonomy proposed in the BMJ by Reader et al. [5].

All complaints were reviewed and categorised accordingly by the authors. Performance data were collected for all patients managed by the ENT Department, either electively or as an emergency, to identify the total number of patients treated over the same time period.

## 3. Results

We identified 251 complaints in total during the set time period. We excluded 9 due to incomplete information (e.g., original complaint letter not available). Of the remaining 242 complaints, 54.1% were about female patients, with the remaining 45.8% relating to male patients. Complaints were related to patients with a mean age of 48.3 years (range 3–98). Furthermore, 11.9% (*n* = 29) were under 18 years, 36.7% (*n* = 89) between 19 and 40 years, 25.2% (*n* = 61) between 41 and 60 years old, and 26% (*n* = 63) over the age of 61 years.

The majority of complaints were filed by patients themselves (*n* = 222), followed by their relatives (*n* = 16). These were most commonly directed at the administrative team (52%, *n* = 126), with the management (23.5%, *n* = 57), the physician (16.9%, *n* = 41), the nursing staff (5.3%, *n* = 13), and allied healthcare professionals (2%, *n* = 5) being the target group for the rest of the complaints, respectively.

Of the 242 complaints, just over half (50.1%) were related to management and administration whilst 25% were about staff-patient relationships and 24.7% were related to clinical care. Furthermore, 61.4% of patients complained about more than one aspect of their care and were allocated multiple themes. Full breakdown of the results is shown in Table 1.

### 3.1. Management

The majority of complaints were regarding management with over a third relating to delays in access to services and treatment (37%, *n* = 200). Many complained that their appointments for clinic or theatre were repeatedly cancelled and postponed, meaning that patients felt that the wait to get an outpatient appointment was too long. As a result, they were concerned that their condition had deteriorated whilst waiting to be seen.

Patients also often complained that they were unable to contact the clinic in order to amend their appointment, or ask for clarification regarding follow-up. Patients described difficulties in contacting the outpatient clinic both by phone and/or by email. Moreover, 10.2% of complaints in this theme (*n* = 55) related to service issues such as staffing, resources, and other institutional issues. These were regarding poor staffing in the outpatient department and overbooking of clinics as well as nonfunctioning equipment (e.g., electrocardiogram and CPAP) and poor food quality.

### 3.2. Quality of Clinical Care

Quality of clinical care was mentioned in 20% of complaints (*n* = 107), and it regarded patient journey, examination, treatment, and miscellaneous quality of care issues. The remainder 4.6% (*n* = 25) concerned safety issues, such as errors in diagnosis, medication prescribing or administration errors, safety incidents and skills, and conduct issues. This shows a change from similar previous studies, which complaints have predominantly heavily featured clinical issues [8].

Quality of care complaints frequently mentioned poor interdepartmental communication, which led to delays in assessment and erroneous clinic appointments. A few patients questioned whether they had received the correct treatment and/or felt that an alternate, better treatment was not offered. Patients have also mentioned confusion regarding preoperative admission plans and discrepancy between the treatments discussed in clinics and the treatments offered on the day of the operation.

Very few complaints regarded an unpleasant examination by healthcare staff in the outpatient setting or poor postoperative outcome. When this did occur, it related to pain and discomfort during minor procedures in the outpatient clinic such as flexible nasal endoscopy or aural microsuctioning.

### 3.3. Safety of Clinical Care

Clinical complaints which related to safety mostly concerned skills and conduct of healthcare staff or slow clinical diagnosis as a result of delayed diagnostic scans. Often, patients felt that staff were rushed and therefore did not listen to their complaints which also related to poor communication and further hindered patient-staff relationships.

When this occurred, there was also a perceived lack of skill whilst being examined by the healthcare professional they encountered. This most frequently related to flexible nasal endoscopy, otoscopy, and audiological assessment, and this meant that some patients were worried about a missed diagnosis.

Whilst individuals were not mentioned in the complaints, complaints referred to doctors of all grades, with no predilection to the level of seniority.

Critical safety incidents were rare; one patient was given the incorrect wrist band and thus misinformed of the information regarding the operation they were scheduled to have, as well as the discharge information. Furthermore, a patient received an erroneous dose of anticoagulation, and there was an incident where scissors were left within reach of a confused patient, unfortunately leading to a percutaneous gastrostomy tube being cut.

### 3.4. Relationships: Communication

Communication has long been a common theme in complaints, and our study directly attributed 15.1% (*n* = 81) of cases to communication breakdown and poor patient-staff dialogue. The rest of the complaints (9.9%, *n* = 53) involved staff attitude, confidentiality, consent, respect and dignity, and abuse. We had no reports of discrimination. The theme of *“communication breakdown”* came up in 6.5% of complaints, with patients mentioning inadequate information regarding diagnosis, treatment, and prognosis.

Complaints regarding patient-staff dialogue are more specific and patients were unhappy with the way they were spoken to and the tone that was being used, leading to a breakdown in patient-staff relationship.

Patients also complained about cancelled clinics which they were not informed of in a timely manner and for not receiving letters from the outpatient department summarising their encounter.

When patients complained about the conduct of healthcare professionals, it most commonly related to the lack of appropriate introduction and lack of apology for the clinic delays. Furthermore, there was confusion regarding the specialty and role of the healthcare professional. This has also been recognised in the “hello my name is…” campaign set up to encourage healthcare professionals to introduce themselves to patients in 2013 [9]. Our NHS Trust is not currently enrolled in this campaign; however, a future comparative study if implemented would be interesting to analyse.

### 3.5. Relationships: Other Issues

Complaints about staff attitude mentioned the same theme of poor or rude communication or unhelpful staff. Certain patients felt that poor staff attitude was reflected in the fact that there were delays in receiving pain relief and in attending to patients' daily needs such as bathing and toileting. There was an isolated case in which a patient felt that the attending staff member preached their own religious beliefs, and a further case of where it was felt that staff dealt with an aggressive patient poorly in the outpatient department.

Very few patients complained about not being treated or spoken to with respect, care, and dignity. Discrimination was not reported in any of the complaints.

## 4. Discussion

The results outline constraints with staffing and increasing service demands, and however issues with delays in access have now become common and are sometimes regarded as inevitable, they can further burden patients and potentially lead to missed medical emergencies. There have been many proposals to reduce clinic demand and improve waiting times, such as attempting to reduce the number of patients failing to attend appointments or using computer-based predictors to assess capacity, but these have not yet been translatable to day-to-day clinical practice. Current strategies unfortunately involve further stretching available resources to fit the needs of more patients, which is not a sustainable model. A new model has been proposed in which clinic appointments are released based on demand rather than predicted capacity; this, however, needs further data regarding reliability and usability of the results [10].

Patient experience has been shown to correlate with quality of care and clinical effectiveness in a recent systematic review across multiple specialties. This further strengthens the case for altering the process of measuring quality in healthcare and advocates adding subjective patient experience to objective measurements of safety and effectiveness [6].

Patients and relatives have a nonmedical view of clinical errors; however, the literature shows a correlation between high-level patient incidents and poor safety of care. Furthermore, complaints can highlight safety issues and near-misses that are not represented in incident report forms. This was the case in the famous Mid Staffordshire NHS Foundation Trust Public Inquiry which brought to light serious patient care issues which had long been flagged up in patient complaints. Following this, the House of Commons Public Administration Committee has advised for improving handling of patient complaints [11]. Despite this, patients and relatives remain an underused resource although there has been a gradual change in attitudes in the NHS.

With regard to hospital correspondence, the NHS Plan 1 was set out in April 2004, obliging NHS Trusts to send patients a copy of all the correspondence between clinicians. More recent guidelines from the Academy of Medical Royal Colleagues in October 2018 have gone further by advising clinicians to avoid using Latin words and acronyms in order to make clinical correspondence more comprehensible to patients [12].

## 5. Conclusion

Collecting and analysing complaints enables us to better assess the perceived quality of healthcare encounters and help improve care. NHS England received over 175000 written complaints between 2013 and 2014, which equals to 479.1 complaints per day. This is, currently, a large underused source of data which could be used to analyse current healthcare provision and failures in patient care [13]. This evidentiates the lack of a formal structured cross-specialty taxonomy for recording complaints which would enable accurate collection as well as comparing healthcare provision across different centres and specialties. Furthermore, this issue has been also flagged up in a recent literature review which reiterated the need for comprehensive and integrated patient complaint systems in order to allow learning from complaints data. This has also noted the poor public awareness of complaints channels and the lack of official standardised training available to staff in regards to complaint management [14].

Patients expect a good encounter to focus on their individual healthcare needs, and person-centred care has shown to positively impact healthcare interactions, outcomes, and patient satisfaction [1]. Furthermore, Eriksson and colleagues argued that communication is the basis of the patient-caregiver relationship and reiterated that inadequate time has a negative impact on this [15]. Improving the efficiency of patient encounters by acting on patient feedback can improve patient outcomes not only by improving the quality of care but also by improving patient engagement with health services and adherence to treatment [16].

Previous studies have found that the majority of complaints in the NHS have focused on communication and were targeted at primary care givers [4, 15, 17]. Our study, however, shows a significant shift; where over a third of complaints related to delays and cancellations and were aimed at management. A recent poll in 2017 showed that patients are now concerned about the effect increasing pressures on the NHS has on “the caring ethos of the NHS” and attributing this to staff being overworked and overstretched [18]. This may be a by-product of a healthcare system under financial and staffing pressures. Patients are waiting longer for both inpatient and outpatient care, and recent initiatives to alleviate winter pressures have seen less urgent elective procedures being cancelled [19].

A study looking at all the operations scheduled across 90% of NHS hospitals in the UK for one week in March 2017 found that 14.2% of operations were cancelled or postponed during that time. Of the 14936 patients who were having elective procedures, 1499 have had the procedure cancelled at least once previously, the most common reasons being insufficient bed capacity (31%) or theatre capacity (12.7%), clinical reasons (33%), and other nonclinical reasons such as staff shortages and equipment failure (23.3%) [20].

When delays or cancellations occurred, patients were often most concerned that failure to be seen may impact on their medical condition, often without specific explanation. Additionally, when they called to clarify, they were unable to get through to a member of staff to enquire further. This, in principle, can be viewed as a communication failure between the healthcare provider and the patient. Providing a reason for the cancellation and informing patients that this delay in management will not impact on the ultimate outcome may reduce anxiety and provide reassurance. This is a technique which has already been employed in the aviation industry to improve customer satisfaction. In 2000, the United States Congress passed the Airline Passenger's Bill of Rights where it set standards for service performance with one key requirement for airlines “To inform passengers accurately and truthfully of the reason for the delay, cancellation, or diversion of a flight”.

## 6. Limitations and Weaknesses

The main limitations and weaknesses of this paper relate to incomplete and retrospective data collection, including the lack of detailed follow-up of how these complaints were managed. Whilst all complaints were assigned as “resolved,” patient satisfaction with the outcome is not assessed. This information would give us more insight into further improving healthcare encounters. A further limitation is the lack of a structured taxonomy when collecting the complaints and the lack of detailed demographics to ensure anonymity.

Moreover, we have only analysed the complaints received by the ENT Department. This has a level of bias associated with it, and these findings might not correlate with other department findings within the same hospital.

It is important to highlight that whilst analysis of complaints information can help guide improvements in patient care, it is impossible to satisfy every patient. For example, whilst we would argue that most patients would find prospective notifications of appointments a positive step; one patient complained that the outpatient department relies on text messages to update patients on clinic appointments.

Some of the complaints in our review were beyond the control of both individual healthcare staff and the provider as a whole, such as need for repeat preoperative assessments and need for further investigations before deciding on treatment. Nonetheless, patients perceived resultant delays as poor service. Future research should look at what defines bad service and the traits, characteristics, and changing expectations of patients. This paper did not evaluate the changing cultural attitudes and service quality expectations.

## 7. Future Improvements

In a world of a health system under increasing pressures, new technology could improve accessibility to healthcare services and reduce unnecessary hospital admission and free resources. For example, the use of telemedicine has been shown to reduce hospital admission in certain age groups but must be considered carefully. Whilst this technology has been viewed as an acceptable alternative to patients, the long-term impact on delivery of healthcare and patient-doctor relationships has yet to be evaluated completely. Furthermore, as technology evolves fast, robust studies need to be carried out frequently to maintain validity and show improvement [21, 22].

Furthermore, as new waves of technology are constantly being introduced, many have considered the role of apps or automated telephone communication systems (ATCS) with regard to improving patient care. As promising as this sounds, these applications still require more work to ensure safety of use as pointed out by a recent Cochrane review [23].

Nonetheless, in the world of increasing strain on healthcare and rapidly advancing technology, patient experience must feature as a central consideration with future changes in healthcare trends. The role of the healthcare provider is to treat the patient as a whole and as Sir William Osler said: “The good physician treats the disease; the great physician treats the patient.” Further work is clearly required in how healthcare organisations collect, analyse, and resolve patient complaints and more emphasis must be put on patient feedback when assessing quality of care. Universal, cross-specialty use of similar coding taxonomies is the next step in accurately and usefully collecting patient experience data, reviewing results in context, and mapping potential trends.

## Figures and Tables

**Table 1:** Summary of complaints categories as per the coding taxonomy.

Complaint type	Percentage (number)
**Clinical**	**24.7% (132)**
Quality	
Examination	2% (11)
Patient journey	5.8% (31)
Quality of care	7.3% (39)
Treatment	4.5% (26)
Safety	
Errors in diagnosis	0.9% (5)
Medication errors	0.1% (1)
Safety incidents	1.1% (6)
Skills and conduct	2.4% (13)
**Management**	**50.1% (268)**
Institutional issues	
Bureaucracy	1.3% (7)
Environment	1.4% (8)
Finance and billing	0.7% (4)
Service issues	4.8% (26)
Staffing and resources	1.8% (10)
Timing and access	
Access and admission	15.9% (85)
Delays	21.5% (115)
Discharges	0.7% (4)
Referrals	1.6% (9)
**Relationships**	**25% (134)**
Communication	
Communication breakdown	6.5% (35)
Incorrect information	3.3% (18)
Patient-staff dialogue	5.2% (28)
Humaneness and caring	
Respect, dignity, and caring	1.8% (10)
Discrimination	0% (0)
Patient rights	
Staff attitude	6.1% (33)
Abuse	0.1% (1)
Confidentiality	0.5% (3)
Consent	1.1% (6)

## Data Availability

The data used to support the findings of this study are available from the corresponding author upon request.
